# Incidence of inflammatory breast cancer in patients with clinical inflammatory breast symptoms

**DOI:** 10.1371/journal.pone.0189385

**Published:** 2017-12-20

**Authors:** Yohann Dabi, Lauren Darrigues, Kelly Pons, Mylène Mabille, Issam Abd alsamad, Rana Mitri, Dounia Skalli, Bassam Haddad, Cyril Touboul

**Affiliations:** 1 Faculté de médecine de Créteil UPEC–Paris XII, Service de Gynécologie-Obstétrique et Médecine de la Reproduction, Centre Hospitalier Intercommunal de Créteil, Créteil–France; 2 Service de radiologie, Centre Hospitalier Intercommunal de Créteil, Créteil–France; 3 Service d’anatomopathologie, Centre Hospitalier Intercommunal de Créteil, Créteil—France; 4 UMR INSERM U965: Angiogenèse et Recherche translationnelle, Hôpital Lariboisière, Paris, France; University of South Alabama Mitchell Cancer Institute, UNITED STATES

## Abstract

**Background:**

To describe a large cohort of women with non-puerperal inflammatory breast and to identify characteristics of inflammatory breast cancer.

**Methods:**

All patients consulting for inflammatory breast syndrome in the breast unit of our tertiary University hospital between September 2013 and December 2015 were prospectively included. We excluded women who were pregnant or in the postpartum period. Patients underwent systematic clinical examination and imaging (breast ultrasonography and mammography). A biopsy was performed if the clinician suspected a malignant lesion of the breast. Clinicopathologic and radiologic data were registered. Statistics were performed using R (3.0.2 version) software.

**Results:**

Among the 76 patients screened and included, 38 (50%) had a malignant lesion at final diagnosis, 21 (27.6%) were diagnosed with infectious disease and 17 (22.4%) with inflammatory disease of the breast. When compared to patients with benign disease, patients with a malignant lesion were significantly older (p = 0.022, CI95% 1.78–14.7), had a significantly bigger palpable mass (p<0.001, CI 95% 22.8–58.9), were more likely to have skin thickening (p = 0.05) and had more suspicious lymph nodes at clinical examination (p<0.001, CI 95% 2.72–65.3). Precise limits on ultrasonography were significantly associated with benign lesions. The presence of a mass (p = 0.04), micro calcifications (p = 0.04) or of focal asymmetry (p<0.001, CI95% 1.3–618) on mammography was significantly associated with malignant disease.

**Conclusion:**

Inflammatory breast cancer was common in our cohort of women consulting for inflammatory breast syndrome. Identifying these patients with high-risk malignancy is crucial in the management of an inflammatory breast.

## Introduction

Inflammatory breast, sometimes referred to as red breast syndrome, is a classic but rare complaint in women consulting at gynecology emergency centers [1, 2]. It usually presents as a red and hot breast and is often associated with breast pain [3, 4].

The spectrum of the possible diagnoses for women presenting with inflammatory breast is wide, ranging from a perfectly benign abscess to malignancy. It is usually classified into one of three main groups: malignant, infectious, or inflammatory without infection (or mastitis) [5]. The malignant form is known as inflammatory breast cancer (IBC), a particularly aggressive form of breast cancer at high risk of metastasis and relapse [6]. Differential diagnosis is therefore crucial in this setting so as not to misdiagnose a malignancy or delay initiation of important treatments: IBC constitutes a therapeutic emergency requiring primary chemotherapy [7, 8].

Most of the literature in this field focuses on infectious mastitis. To date, few cohorts of inflammatory breast disease have been reported [1, 5] and most of these include postpartum breast inflammatory diseases, which may bias clinical description and consequent management.

The objective of our study was to describe the diagnostic workup associated with nonpuerperal inflammatory breast in a large cohort and define the characteristics associated with IBC.

## Patients and methods

### Details of ethics approval

Our study was non interventional. The CERES approved the realization within our centre of an observational study not modifying patients’ management.

Every patients consulting for an inflammatory breast during our inclusion period was informed the data collected might be used for medical and / or research purpose and gave oral consent. Written consent was not required nor asked as the study was non interventional. All patients were sent information letters offering them the possibility of refusing to have their data included.

All clinical investigation has been conducted according to the principles expressed in the Declaration of Helsinki.

### Patients

All consecutive patients referred for inflammatory breast to the Breast Unit of our tertiary University hospital department of Obstetrics and Gynecology between September 2013 and December 2015 were prospectively included. Inflammatory breast was defined as a red and hot breast often associated with pain [3, 4]. All relevant clinical and imaging data were recorded at the time of inclusion. We excluded women who were pregnant or in the postpartum period.

All patients were initially examined by a gynecologic oncologist. Clinical examination consisted of breast palpation and investigation for pathologic lymph nodes. Imaging, including ultrasonography of the breast and the axilla associated with a bilateral mammography when feasible, was done for all included patients. Most imaging exams were performed and interpreted in the Radiology Department of the hospital by breast imaging radiologists. Some women had already been assessed at another center at the time of the consultation in which case their imaging exams were reviewed by our breast radiologists if deemed necessary. Women who were in considerable pain were immediately put under treatment and their mammography postponed.

### Medical strategy and management

Patients diagnosed with inflammatory breast were managed according to the latest French guidelines [9–11]. S1 Fig reproduced the decisional tree for the management of inflammatory breast patients.

Patients diagnosed with a suspicious lesion, either at clinical examination or on imaging, underwent breast and punch skin biopsy. Therefore, in the context of inflammatory breast, the diagnosis of IBC was based on the discovery of breast cancer at biopsy +/- infiltration of cancer cells within dermal tissue when a punch biopsy was performed. In the case of advanced malignancy, fluorodeoxyglucose positron emission tomography–computed tomography.

(FDG^18^ PET CT) and Chest computed tomography (CT) were performed to detect extra-mammary invasion. Patients were also tested for the breast tumor marker CA 15.3 with a cut off of above 25 being considered positive.

Breast biopsy was also performed in women with non-infectious inflammatory breast to characterize the lesion.

An antibiotic test-and-treat strategy was systematically applied for patients without any sign of breast cancer at clinical examination. The choice of antibiotics was based on French recommendations and mainly targeted *Staphylococcus aureus* sp. All the women underwent a second clinical evaluation 2 weeks after initiation of antibiotic therapy. Persistent symptoms were an indication for breast or skin biopsy and further imaging including magnetic resonance imaging (MRI) was discussed.

Surgery or percutaneous drainage under ultrasound guidance was performed for all patients with infectious breast disease if there was no favorable response 2 weeks after initiating the antibiotic test-and-treat strategy. Surgery classically consisted of conventional incision and introduction of a mesh into the wound. If there was per-operative suspicion of malignancy, a breast biopsy was performed.

After appropriate management and complementary exams, the women were classified into one of three groups based on the “final diagnosis”: infectious, inflammatory or malignant. The final diagnosis was used to analyse the relevance of the imaging exams performed.

Statistical analysis was based on the Student’s t test for continuous variables and the χ^2^ test or Fisher’s exact test for categorical variables. The p-value expressed in our study results from a comparison of patients with a malignant breast lesion with patients with a non-malignant lesion (*i*.*e*., inflammatory or infectious). Values of p < 0.05 were considered to denote significant differences. Data were managed with an Excel database (Microsoft Corporation, Redmond, WA, USA) and analyzed using R (3.0.2 version) software, available online.

## Results

### Patient management

**Fig 1:**
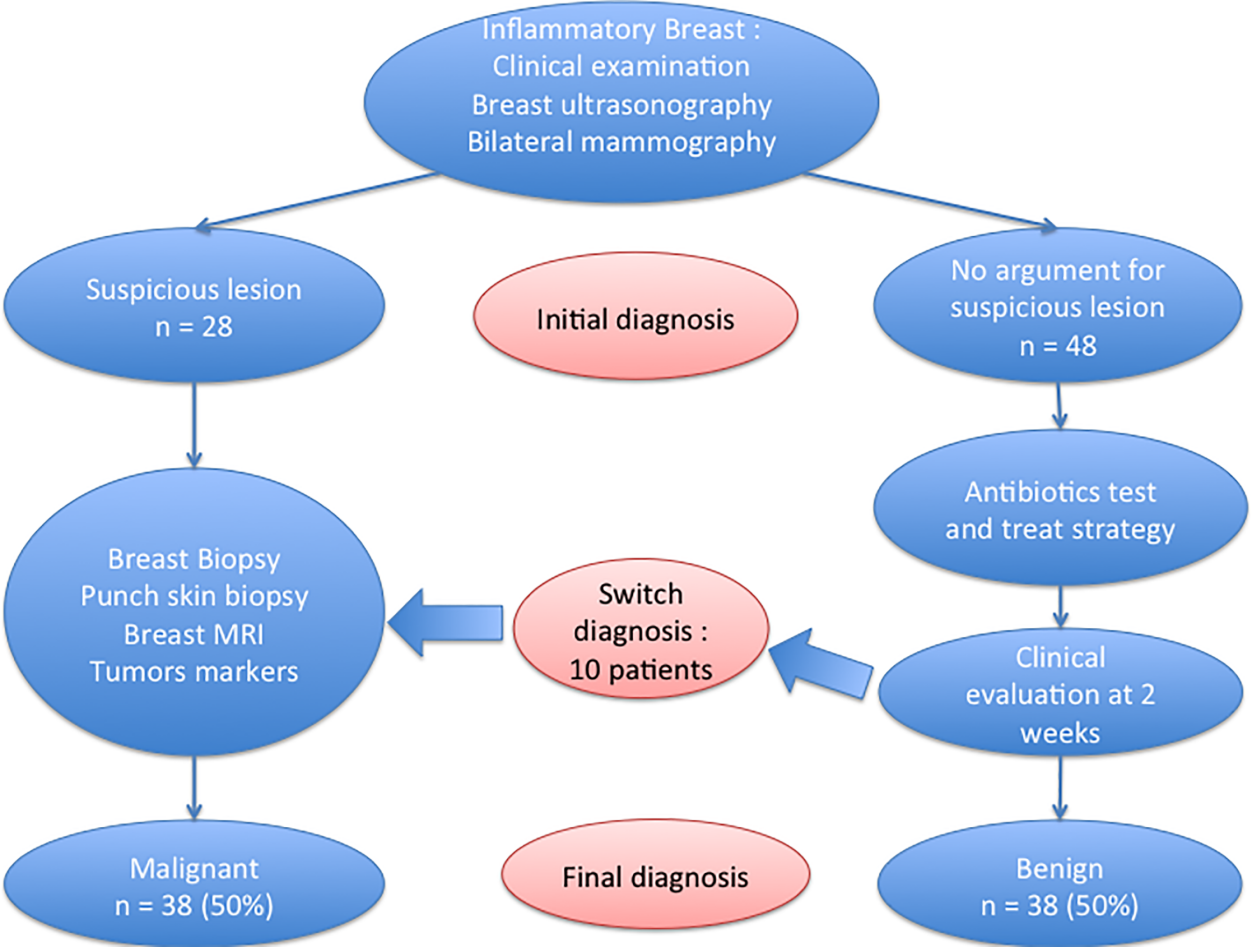
Flow chart of the study.

A total of 76 patients were screened and included for analysis. Among them, 21 (27.6%) were diagnosed with an infectious disease of the breast, 17 (22.4%) with a benign inflammatory disease and 38 (50%) with breast cancer (Fig 1).

Forty-six patients (96% of patients without initial suspicion of malignancy) underwent an antibiotic test-and-treat strategy. Among the patients with benign disease, seven (18.4%) underwent surgical drainage after failure of the antibiotic test-and-treat strategy. Respectively 10 (36%) and nine (53%) patients in the infectious and inflammatory groups underwent breast biopsy, mainly to confirm diagnosis and adapt the choice of antibiotics when indicated.

**Fig 2:**
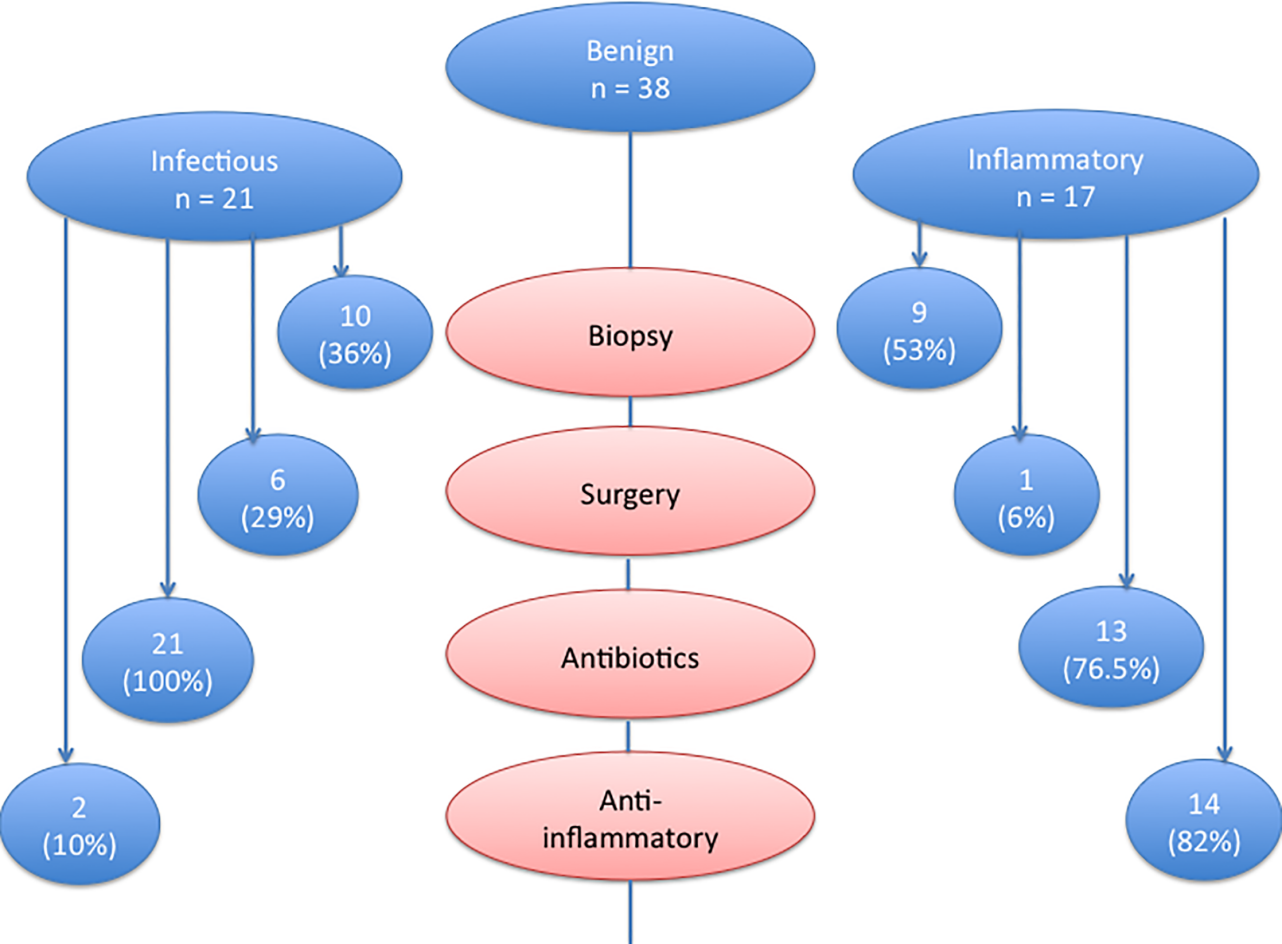
Diagnostic and treatments procedures in patients with benign breast disease.

Patients with an infectious disease of the breast were mostly diagnosed with retroareolar abscess (n = 15, 71.4%). Other diagnoses retained in this group were infected galactoceles and infected cysts (n = 4, 19%), an abscess associated with a fibroma (n = 1, 4.8%), and a posttraumatic fat necrosis with recurrent abscess (n = 1, 4.8%). All patients with infectious disease of the breast were administered antibiotics which were combined with an anti-inflammatory drug in two (11.8%). Six of the women (35.3%) underwent surgery after failure of the antibiotherapy (Fig 2).

Patients with inflammatory disease of the breast were diagnosed with periductal mastitis (n = 5, 29.4%), post irradiation mastitis (n = 3, 17.6%), granulomatous mastitis (n = 2, 11.8%), Mondor disease (n = 1, 5.9%), post-traumatic fat necrosis (n = 1, 5.9%), inflammatory adenofibroma (n = 1, 5.9%) or unclassified mastitis (n = 4, 23.5%). Thirteen of these patients (76.5%) were treated with antibiotics and 14 (82.4%) with anti-inflammatory drugs. One of the patients (4.8%) with granulomatous mastitis underwent breast surgery after failure of both antibiotherapy and anti-inflammatory treatment (Fig 2).

Among the 38 patients diagnosed with IBC, 23 (60.5%) were tested for CA 15.3 with a mean value of 92.7 U/mL (SD 25–103). They all underwent breast biopsy to confirm the diagnosis and characterize the tumor. Twenty-seven (71.1%) of these patients had advanced disease with pathologic lymph nodes and 11 (26.3%) had multiple metastases at diagnosis. Twenty-four patients had grade III disease and 13 had positive hormone receptors. Seven patients were HER2-positive. The mean Ki67 in patients diagnosed with IBC was 43% (IQ 16–70%).

### Patients’ characteristics and clinical examination findings

The main characteristics of the patients are presented in Table 1. We compared the clinical and radiological features of patients with IBC and those without. Patients with IBC were significantly older (p = 0.022), less painful (p = 0.03), and were less likely to have fever (p = 0.027). They also had a significantly bigger palpable mass than those with benign disease: 81.3 mm vs 41.3 mm (p < 0.001). Skin thickening was also more frequent in this group: 21 (58.3%) vs 10 (32.3%) (p = 0.05) as were suspicious lymph nodes: 19 (50%) vs 3 (8.1%) (p<0.001). (Table 2)

**Table 1:** Clinical characteristics of the patients.

Characteristics	Number of patients Total = 76	Missing Data	Infectious (n = 21)	Inflammatory (n = 17)	Malignant (n = 38)	p—value
**Age (in years)**	48.9	0	44.2 (IQ 40–54.2)	46.2 (IQ 40–54)	52.8 (42–55)	***0*.*022*** ***(CI95% 1*.*78–14*.*7)***
**History of breast feeding:**	26 (34.2%)	13	5 (23.8%)	6 (35.3%)	15 (39.5%)	0.27
**Tobacco use**	19 (25%)	16	5 (23.8%)	6 (35.3%)	8 (21.1%)	0.46
**Personal history of invasive breast cancer**	11 (14.5%)	0	0	4 (23.5%)	7 (18.4%)	0.51
**Personal history of infectious breast disease**	12 (15.8%)	0	4 (19.0%)	4 (23.5%)	4 (10.5%)	0.34
**Personal history of dermatological disease**	2 (2.6%)	0	0	2 (11.8%)	0	/
**Family history of invasive breast cancer, 1**^**st**^ **or 2**^**nd**^ **degree**	22 (28.9%)	3	6 (28.6%)	4 (23.5%)	12 (31.6%)	0.85
**Symptoms at the time of the consultation**						
*Pain*	44 (57.9%)	0	16 (76.2%)	11 (64.7%)	17 (44.7%)	***0*.*03***
*Perception of a mass*	54 (71.1%)	0	18 (85.1%)	6 (35.3%)	30 (78.9%)	0.2
*Ulceration*	13 (17.1%)	0	2 (9.5%)	1 (5.9%)	10 (26.3%)	0.06
*Breast discharge*	13 (17.1%)	0	4 (19%)	3 (17.6%)	6 (15.8%)	1
*Fever (> 38*.*5°C)*	12 (15.8%)	0	4 (19%)	6 (35.3%)	2 (11.1%)	***0*.*027***
*Impaired general condition*	6 (7.9%)	0	0	1 (5.9%)	5 (13.2%)	0.199

Data are given as mean (interquartile range) or n (%)

**Table 2:** Physical examination.

Characteristics	Number of patients Total = 76	Missing data	Infectious (n = 21)	Inflammatory (n = 17)	Malignant (n = 38)	p—value
**Mean size of the lesion (in mm)**	62	16	41.2mm		83.1mm	***<0*.*001 (CI95% 22*.*8–58*.*9)***
*< 30 mm*	18 (23.7%)		10 (47.6%)	4 (23.5%)	4 (10.5%)	
*30 < X < 60 (mm)*	18 (23.7%)		5 (23.8%)	5 (29.4%)	8 (21%)	
*> 60 mm*	23 (30.3%)		3 (14.3%)	2 (11.8%)	18 (47.4%)	
Other unusual coloration*	9 (11.8%)	2	1 (4.8%)	1 (5.9%)	7 (18.4%)	0.15
**Breast Pain**	42 (55.3%)	0	15 (71.4%)	13 (76.5%)	14 (36.8%)	***0*.*002***
**Sensitivity loss**	9 (11.8%)	6	0	2 (11.8%)	7 (18.4%)	0.15
**Skin thickening**	31 (40.8%)	9	3 (14.3%)	7 (33.3%)	21 (55.3%)	***0*.*05***
**Consistency of the skin**		6				/
*Indurated*	48 (63.2%)		8 (38.1%)	10 (58.8%)	28 (73.7%)	
*Tender*	8 (10.5%)		3 (14.3%)	5 (29.4%)	2 (5.3%)	
*Fluctuating*	10 (13.2%)		8 (38.1%)	1 (5.9%)	2 (5.3%)	
*Necrotic*	3 (3.9%)		0	0	3 (7.9%)	
**Perception of a mass**	34 (44.7%)	0	11 (52.4%)	4 (23.5%)	19 (50%)	0.48
**Areolar skin changes (retraction)**	17 (22.4%)	0	4 (19%)	3 (17.6%)	10 (26.3%)	0.58
**Palpable cord**	1 (1.67%)	1	0	1 (5.9%)	0	/
**Suspect lymph node**	22 (28.9%)	1	2 (9.5%)	1 (5.9%)	19 (50%)	***<0*.*001 (CI95% 2*.*72–65*.*3)***

* Other unusual coloration included: black / necrotic (2 patients) and purple (7 patients)

### Ultrasonography findings

The results of all the 68 patients who underwent ultrasonography were available for analysis (Table 3). The remaining 8 patients did not undergo ultrasonography as they were diagnosed with IBC by per-operative breast biopsy. The only sign significantly associated with benign disease was precise limits of the mass when present: 19 (87%) vs 6 (27.3%) (p<0.001). As for the clinical exam, presence of a suspicious lymph node on ultrasonography was highly suggestive of IBC. All other parameters usually associated with either benign or malignant disease were not statistically different between the groups.

**Table 3:** Ultrasonography comparison.

Characteristics	Number of patients (n = 68)	Missing Data	Infectious (n = 20)	Inflammatory (n = 17)	Malignant (n = 31)	p—value
**Presence of a mass**	45 (66.2%)	1	15 (75%)	8 (47.1%)	22 (75.9%)	0.40
**Echo of the mass**		4				/
*Anechoic*	4 (9%)		3 (20%)	1 (12.5%)	0 (*)	
*Hyperechoic*	10 (22.7%)		6 (40%)	1 (12.5%)	3 (13.6%)	
*Hypoechoic*	27 (61.4%)		6 (40%)	6 (75%)	15 (68.2%)	
**Mean size of the mass (in mm)**		4	14 patients: 31.1	8 patients: 31.6	19 Patients: 33.2	0.86
**Precise limits**	25 (56.8%)	1	11 (73.3%)	8 (100%)	6 (27.3%)	***< 0*.*001***
**Hyperechogenicity of the fat**	24 (36.4%)	36	6 (30%)	10 (58.8%)	8 (25.8%)	1
**Interstitial edema**	16 (24.2%)	15	3 (15%)	6 (35.3%)	7 (22.6%)	1
**Retroareolar duct dilatation**	7 (10.6%)	14	3 (15%)	1 (5.9%)	3 (9.7%)	/
**Skin > 2 mm of thickness**	22 (33.3%)	15	6 (30%)	6 (35.3%)	10 (32.3%)	0.89
**Mammary duct fistula**	1 (1.5%)	11	1 (5%)	0	0	/
**Hypervascularization**	8 (12.1%)	13	2 (10%)	1 (5.9%)	5 (16.1%)	
**Suspicious lymph node**	9 (13.6%)	7	0	0	9 (29%)	/
**Cyst**	8 (12.1%)	2	5 (25%)	1 (5.9%)	2 (6.5%)	/
**Abscess**	13 (19.7%)	1	11 (73.3%)	2 (11.8%)	0	/
**Size of the abscess** **(in mm)**		0	31 (10 patients)	25 (2 patients)	0	/

* 3 patients with missing data

### Mammography findings

Records of the mammography findings were available for 45 patients of our cohort (Table 4). The presence of a mass (p = 0.04), of micro calcifications (p = 0.04) or of a focal asymmetry (p<0.001, CI95% 1.3–618) were significantly associated with IBC. Patients with infectious disease of the breast were more likely to have a mass detected, visible opacity, or spread micro calcification on mammography than those with inflammatory breast disease.

**Table 4:** Mammography comparison.

Characteristics	Number of patients (n = 45)	Missing data	Infectious (n = 11)	Inflammatory (n = 11)	Malignant (n = 23)	p—value
**Presence of a mass**	21 (44.4%)	2	4 (36%)	2 (18.2%)	15 (60.9%)	***0*.*04***
**Mean size of the mass (in mm)**		7	17.6 (3 patients)	3 (1 patient)	49.2 (10 patients)	0.11
**Precise limits of the mass**	7 (35%)	0	1 (25%)	2 (100%)	4 (28.6%)	0.8
**Opacity**	20 (44.4%)	7	6 (54.5%)	2 (18.2%)	12 (52.2%)	0.52
**Presence of calcifications**	20 (44.4%)	2	3 (27.3)	3 (27.3%)	14 (60.9%)	***0*.*04***
**Focal Asymmetry**	9 (20%)	5	1 (9.1%)	0	8 (34.8%)	***< 0*.*001*** ***(CI95% 1*.*3–618)***
**Spread Hyperdensity**	16 (35.6%)	5	5 (45.5%)	3 (27.3%)	8 (34.8%)	1
**Retroareolar duct dilatation**	3 (6.7%)	4	0	2 (18.2%)	1 (4.3%)	/
**Skin > 2 mm of thickness**	9 (20%)	5	1 (9.1%)	1 (9.1%)	7 (30.4%)	0.06
**Suspicious lymph node**	6 (13.3%)	3	1 (9.1%)	0	5 (21.7)	0.18

Including 1 post operative

### Breast MRI findings

Since breast MRI was performed mainly exclusively for some of the patients with a high suspicion of IBC (n = 16, 32%), we did not compare these findings with patients with benign lesion of the breast. A third of the patients with IBC had skin thickness or interstitial edema and 25% had hyperdensity of the fat around the lesion. Presence of a mass and suspicious lymph nodes were respectively present in 12 (75%) and 11 (68.8%) patients with IBC (Table 5)

**Table 5:** MRI analysis.

Characteristics	Number of patients (n = 20)	Missing data	Infectious (n = 1)	Inflammatory (n = 3)	Malignant (n = 16)
**Presence of a mass**	13 (65%)	1	1	0	12 (75%)
**Size of the mass**	12 (60%)	1	*	0	48.2 mm
**Precise limits**	3 (15%)	1	*	0	3 (20%)
**Hyperdensity of the fat**	4 (20%)	9	0	0	4 (25%)
**Interstitial edema**	8 (40%)	7	0	2 (66%)	6 (37.5%)
**Hypervascular lesion**	7 (35%)	7	0	0	7 (43.8%)
**Non-mass like enhancement**	7 (35%)	6	1	1 (33%)	5 (31.3)
**Local enhancement**	12 (60%)	4	1	1 (33%)	10 (62.5%)
**Retroareolar duct dilatation**	2 (10%)	5	0	1 (33%)	1 (6.3%)
**Skin > 2 mm of thickness**	7 (35%)	7	0	2 (66%)	5 (31.3%)
**Washout**	1 (5%)	10	0	0	1 (6.3%)
**Fistular tract on MRI**	1 (5%)	6	0	0	1 (6.3%)
**Suspicious lymph node**	12 (60%)	2	0	1 (33%)	11 (68.8)

* Missing data

## Discussion

In this largest occidental analysis of women presenting with nonpuerperal inflammatory breast syndrome, we found that half of the patients had IBC. While clinical and radiological features may help initial diagnosis, they are non-specific and follow-up and biopsy are mandatory in case of persisting inflammatory breast despite treatment.

This high rate of IBC is an important finding especially as it contrasts with two previous main studies reporting inflammatory breast syndrome [1, 5]. In 2009, Kamal et al. [5] found that 5.6% of patients had IBC in a cohort of 197 patients with mastitis. Similarly, Froman et al. [1] reported a rate of 4.5% of IBC in a cohort of 23 patients presenting with red breast syndrome after screening more than 3700 women presenting at their breast unit over a 2-year period. The first probable explanation for such a difference is that, unlike these two studies, we excluded women who were pregnant or in the postpartum period from our cohort. Inflammatory breast during breastfeeding is common and does not represent a challenge either for diagnosis or for management. Excluding patients with mastitis during breastfeeding was consistent with our main goal to improving management of women suffering with inflammatory breast syndrome. We believe that the high rate of IBC we report here is probably closer to what clinicians may encounter in everyday practice.

The clinical distinction between true IBC and neglected breast cancer progression may prove delicate in some cases. This distinction is clinically relevant since the treatment strategy might differ. In our cohort, we cannot exclude that some of the cases of IBC were in fact cases of neglected cancer progression. Some authors report clinical diagnostic differences between these two scenario that are easily picked up on during physical examination [7, 12–14]. For example, patients with IBC may experience a rapid increase in breast volume almost simultaneously with the skin changes which are highly evocative of IBC: in our cohort, skin thickening and indurated skin was observed in 55% and 73% of women with IBC, respectively. Most patients with IBC do not present a palpable mass on physical examination [14]. In our cohort, however, the most relevant symptom suggestive of malignancy at the time of the consultation was the perception of a mass (78.9%) on physical examination. Therefore, while this sign may be unusual in IBC, any perception of a mass by the patient or the clinician warrants further investigation including systematic breast or skin biopsy in case of doubt.

Breast ultrasonography and a mammography were particularly useful diagnostic tools in our cohort. On ultrasonography, the only factor significantly associated with IBC was the presence of precise limits of the mass. Other authors report similar results [5, 15]. However, a normal ultrasonography does not in itself rule out IBC. Indeed, Gunhan et al. [16] reported absence of a mass in 20% of authentic cases of IBC and no skin changes in 4% of those cases. Mammography is usually less specific to differentiate between mastitis and IBC. Crowe et al. [17] reported that the most relevant sign of IBC was diffuse skin thickening. Mammography was highly relevant in our cohort. As mentioned before, the presence of a mass, of calcifications or of a focal asymmetry were significantly associated with a malignant lesion though not specific of IBC. Several authors have assessed the performance of MRI for the diagnosis of IBC [16, 18–20]. This exam is usually prescribed after ultrasonography and mammography in case of doubt. Sixteen patients with IBC underwent a breast MRI in our cohort. Presence of mass, local enhancement and suspicious lymph nodes were strongly evocative of a malignant lesion.

While some authors [1, 3] have developed algorithms for the management of inflammatory breast, we believe that the need for such an algorithm is questionable. The main issue at hand is to rapidly identify all cases of IBC, a particularly aggressive cancer, in women presenting with inflammatory breast syndrome. Unfortunately, our data were limited regarding the time lapse between first clinical contact and the diagnostic of an IBC in our cohort and we were not able to assess the efficiency of the management of such patients using French guidelines. Once IBC is identified, it should be treated as an emergency. Our efforts should thus focus on correctly identifying these patients at high risk of metastasis and progression. We have identified factors strongly associated with IBC which would allow clinicians to diagnose IBC in women presenting with inflammatory breast syndrome.

## Conclusion

IBC is prevalent in women presenting with non-puerperal inflammatory breast syndrome and should be the major concern of clinicians managing these patients. Clinical and imaging examination may be helpful for this purpose but further investigation by breast biopsy should be undertaken if the slightest doubt remains. We are currently developing a nomogram based on our results to identifying these high-risk patients with an aim to accelerate appropriate management in this emergency setting.

## Supporting information

S1 Fig Decisional tree for the management of patients with inflammatory breast (adapted from Touboul C, Laas E, Rafii A. [Exploration of breast inflammation excluding pregnancy and breastfeeding: Guidelines]. J Gynecol Obstet Biol Reprod (Paris). 2015 Dec;44(10):913–20.).
(PDF)
