# Supplementary figures and images for: Incidence of inflammatory breast cancer in patients with clinical inflammatory breast symptoms
Source: PLoS One. 2017 Dec 20;12(12):e0189385. doi: 10.1371/journal.pone.0189385 (PMC5738061; doi:10.1371/journal.pone.0189385)

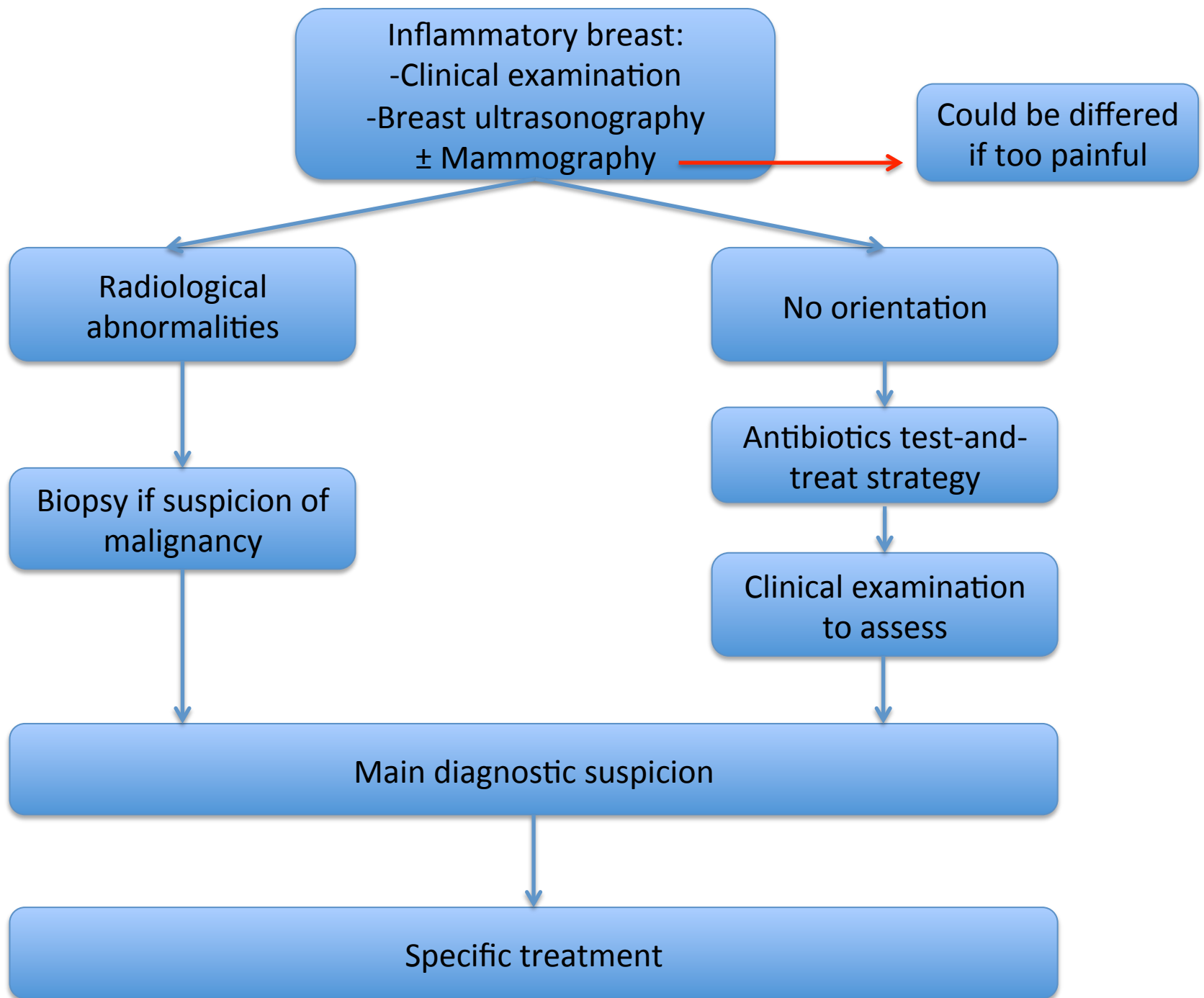

Supplement: S1 Fig — [Exploration of breast inflammation excluding pregnancy and breastfeeding: Guidelines]. J Gynecol Obstet Biol Reprod (Paris). 2015 Dec;44(10):913–20.). (PDF) [file pone.0189385.s001.pdf]
